# Widening the Pool of Factors: Studies Needed to Assess Asthma–Swimming Link

**DOI:** 10.1289/ehp.117-a162a

**Published:** 2009-04

**Authors:** Angela Spivey

Several epidemiologic studies have suggested an association between childhood asthma and exposure to disinfection by-products (DBPs) in the swimming pool environment. In August 2007 a group of clinicians, epidemiologists, exposure scientists, pool operations experts, and analytical chemists met to discuss the literature on childhood asthma and swimming pools, and to develop recommendations for future research. In a review based on the results of that workshop, the authors state that current evidence, while suggestive, is inconclusive for an association with childhood asthma, and they point to several substantial data gaps that must be filled **[*EHP*** **117:500–507; Weisel et al.]**.

The authors articulate several variables that must be measured in more detail to properly characterize inhalation exposure to chemicals around pools. The review calls for a comprehensive assessment of a substantially larger number of chemicals in the pool area than the limited number of DBPs studied to date. Earlier epidemiologic studies suggested trichloramine as a DBP of interest, but one 2007 study revealed previously unknown volatile DBPs in the air surrounding swimming pools.

The frequency and extent of exposure to chemicals around pools also must be studied. In research to date, only simple exposure indices have been used, including whether the pool was indoors or outdoors, specific disinfection treatment, whether the child swam in or was simply present at an indoor pool, and cumulative duration of swimming. But to evaluate the breathing rate and DBP dose delivered to the lungs, more detailed, validated assessments of activity levels are needed. To obtain these data, the authors recommend that future studies use prospective questionnaires in which participants report their pool use and activity levels over time as they occur.

The authors also point to the need for studies that define asthma cases in a rigorous, reproducible way, utilizing the International Study of Asthma and Allergy in Children questionnaire. Previous studies have often used clinical diagnoses, but this may be insufficient for epidemiologic studies because asthma is a heterogeneous disease with no single reliable diagnostic test. Additional needs include development and validation of new biomarkers for asthmatic reactivity and studies designed to refine guidelines for proper pool maintenance and disinfection to reduce levels of DBPs.

The authors conclude this research area requires studies across multiple disciplines. Once chemicals of interest are identified, studies of the mechanisms behind the possible association—such as oxidative stress, inflammation, and changes in lung permeability—may be useful. But long-range prospective studies starting in early childhood will be needed to better gauge the relationship between swimming pools and childhood asthma.

Absent conclusive studies, the authors say children’s exposures should be minimized. Pool managers must be well educated about pool chemistry so they can understand the potential dangers of disinfectants and DBPs. Swimmers, too, must be educated about the need for proper pool hygiene (for example, showering before swimming and not urinating in the pool), as swimmer hygiene can affect the formation of DBPs and the amount of disinfectant used.

## Figures and Tables

**Figure:**
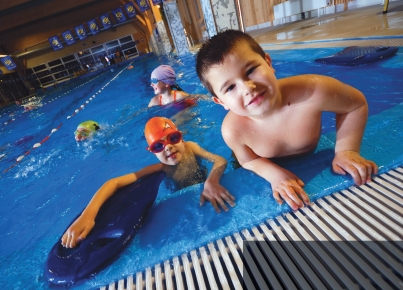
A number of variables still must be studied in greater detail to better characterize exposure to pool-related chemicals.

